# Mechanical plus oral bowel preparation with paromomycin and metronidazole reduces infectious complications in elective colorectal surgery: a matched case-control study

**DOI:** 10.1007/s00384-021-03931-9

**Published:** 2021-04-25

**Authors:** Matthias Mehdorn, Christoph Lübbert, Iris F. Chaberny, Ines Gockel, Boris Jansen-Winkeln

**Affiliations:** 1 Department of Visceral, Transplant, Thoracic and Vascular Surgery, Leipzig University Hospital, Liebigstr. 20, D-04103 Leipzig, Germany; 2 Division of Infectious Diseases and Tropical Medicine, Department of Medicine II, Leipzig University Hospital, Liebigstr. 20, D-04103 Leipzig, Germany; 3 Interdisciplinary Center for Infectious Disease (ZINF), Leipzig University Hospital, Liebigstr. 20, 22 04103 Leipzig, Germany; 4 Institute of Hygiene, Hospital Epidemiology and Environmental Medicine, Leipzig University Hospital, Liebigstr, 22 04103 Leipzig, Germany

**Keywords:** Surgical site infections, Antibiotic bowel preparation, Mechanic bowel preparation, Colorectal resections, Paromomycin

## Abstract

**Purpose:**

Infectious complications are as high as 30% in elective colorectal surgery. In recent years, several studies have discussed the topic of preoperative bowel decontamination prior to colorectal surgery in order to reduce postoperative infectious complications and have found significant effects of oral antibiotic administration with a large variety of drugs used. No study has evaluated the combination of oral paromomycin and metronidazole in this context.

**Methods:**

We performed a prospective single-center study with a matched-pair retrospective cohort to evaluate postoperative infectious complications (superficial site infections, organ space abscess, anastomotic leakage) in elective colorectal surgery.

**Patients:**

A total of 120 patients were available for study inclusion; 101 gave informed consent and were included. A total of 92 patients were matched and subsequently analyzed. We could show a reduction in overall infectious complications in the intervention group (15.2% vs 30.8%, *p* = 0.018; odds ratio 0.333, 95% CI 0.142–0.784) as well as a reduction in superficial surgical site infections (8.7 vs 19.6%, *p* = 0.041, OR 0.333, 95% CI 0.121–0.917). The frequency of the other infectious complications such as intraabdominal abscesses and anastomotic leakage showed a tendency towards decreased frequencies in the intervention group (OR 0.714, 95% CI 0.235–2.169 and OR 0.571; 95% CI 0.167–1.952, respectively). Finally, the oral antibiotic administration led to an almost significantly reduced length of stay (12.24 days vs 15.25 days; *p* = 0.057).

**Conclusions:**

Oral paromomycin and metronidazole with intravenous ertapenem effectively reduce infectious complications in elective colorectal surgery.

**Trial registration:**

The study was registered at Clinicaltrials.gov (NCT03759886) December 17, 2018

## Introduction

Colorectal surgery is an operation prone to complications, in which up to 30% of all cases involve infectious complications such as anastomotic leakage, organ space abscesses, and superficial surgical site infections being present in up to 30% of all cases [1, 2].

In recent years, there has been an ongoing discussion over the past years about the benefits of oral antibiotics (OA) with or without mechanical bowel preparation (MBP) on infectious postoperative complications following colorectal resections. Over 90% of US colorectal surgeons apply mechanical as well as decontaminating bowel preparation [3]. Based on literature review, there have been recommendations to omit MBP [4] as an analysis of existing data in 2010 could not provide benefits on surgical site infections or anastomotic leakage in open colorectal surgery. Large-scale data analysis from the USA, taken from the ACS-NSQIP, has shown that MBP plus oral antibiotics prior to elective colorectal surgery was able to reduce overall surgical site infections by about 50% (14.7% vs 6.2%) [5]. Several other studies have based their analyses on this particular registry with different aspects highlighted, but all judged antibiotic preparation beneficial [5–12]. In general, a protective effect could be found in open and laparoscopic procedures [13]. A similar protective effect of oral antibiotic bowel preparation (OABP) without MBP could be shown in a meta-analysis that tried to point out the importance of OABP only for bowel preparation [14]. Recently, a large randomized controlled trial from Spain promoted the use of OABP without MBP to reduce SSIs in colorectal surgery [15].

In general, different antibiotic regimens are used in accordance with local availability of drugs and possible side effects. Most of the studies report the use of either an aminoglycoside (i.e., neomycine [16–18], kanamycine [19], streptomycin [20], gentamicine [21]) or a fluoroquinolone (levofloxacine [1] or ciprofloxacine [15]) plus metronidazole. Additionally, in all present studies, single-shot antibiotics were used preoperatively, mostly consisting of an IV application of a second-generation cephalosporine plus metronidazole intravenously. The ideal decontaminating agent remains in the bowel after oral ingestion. In Germany, most of the aminoglycosides used in other studies are only available as topic agents but not in an oral formulation designed for intestinal effects. An old but still common aminoglycoside is paromomycin which is used as a soluble powder approved for the prevention of hepatic encephalopathy and which is available in Germany. The only study that evaluated paromomycin for bowel decontamination in colorectal surgery is about 40 years old but already provided evidence for the possible use in colorectal surgery to reduce surgical site infections [22].

Since no data is available on the combined use of paromomycin and metronidazole for preoperative intestinal decontamination in colorectal surgery to prevent infectious complications, we conducted this prospective case-control study with matched-pair analysis.

The aim was to show a significant reduction of postoperative infectious complications by using paromomycin and metronidazole preoperatively after mechanical bowel preparation in colorectal surgery.

## Materials and methods

### Patients and methods

This clinical trial was implemented as a prospective, non-randomized case-control study with matched-pair analysis of a retrospective cohort at the University Hospital Leipzig, Leipzig, Germany. Approved by the local ethics’ committee of the Medical Faculty of the University of Leipzig (010/19-ek), the study was registered at Clinicaltrials.gov (NCT03759886). Written informed consent was obtained from all patients involved. Inclusion criteria were all elective colorectal resections performed between January 2019 and January 2020, who could perform the assigned bowel preparation regime. Exclusion criteria were allergies against the used drugs, emergency surgery, obstructive bowel disease, and patient age under 18 years.

The study protocol for the intervention group (=IG) was as follows: during the afternoon of the day before surgery, patients had to drink 2l of MOVIPREP® solution as MBP (polyethyleneglycol; Norgine, Wettenberg, Germany) within a 2-h time frame. Directly after oral intake of MBP, all patients took 4-g paromomycin and 1-g metronidazole (MBP + OA) each as a single dose without any repetitive dosing. Additionally, all patients received a perioperative single shot for antibiotic prophylaxis with 1-g ertapenem IV within 60 min of skin incision. Due to the half-life of ertapenem, no second dose, even for longer operations, is needed.

After conclusion of the estimated study period in January 2020, we selected a retrospective matched cohort (control group (CG)) from the electronic patient charts with the same inclusion and exclusion criteria from our institution, who had undergone colorectal surgery between 1 January 2017 and 31 December 2018 to perform the statistical analysis to test for sufficient study power (see below). These patients underwent MBP with MOVIPREP® only during the afternoon of the day before surgery. The perioperative single-shot antibiotic prophylaxis within 60 min from skin incision consisted of 1.5-g cefuroxime plus 500-mg metronidazole. A second dose of intravenous antibiotics was given 4 h after the initial dose if the duration of surgery was longer than 4 h.

The following matching parameters were used with descending importance during the matching process to avoid inadequate matching: OPS code, diagnosis, age, BMI, and ASA score. For BMI, we accepted an age difference of ±5 kg/m^2^, for age ±5 years. For ASA, we matched ASA III accordingly but ASA I or II interchangeably. If there was no matchable ASA III patient, comorbidities were evaluated for an equally comorbid ASA II patient as ASA is a nonetheless subjective parameter, set by the evaluating anesthesiologist.

**Fig. 1:**
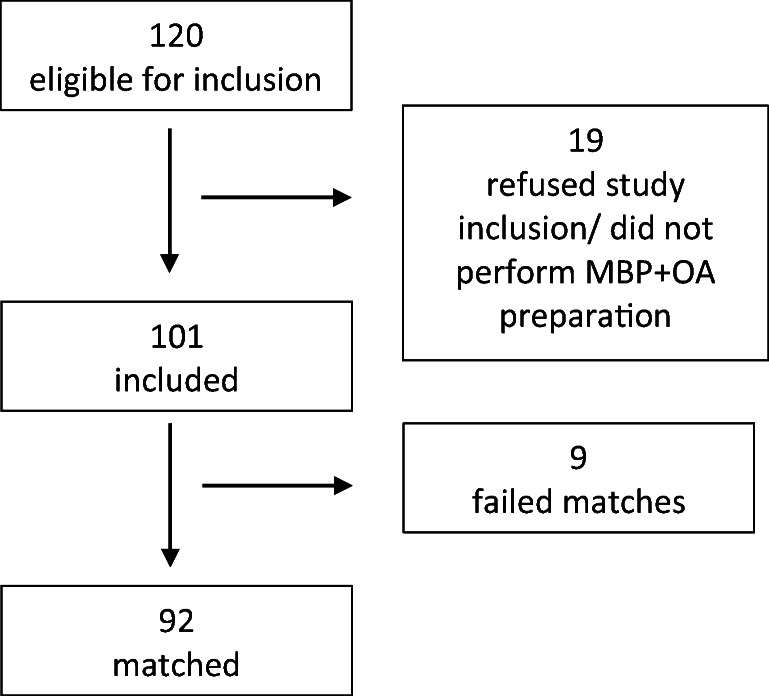
The selection process

All patients that had MBP + OA intake for more than 1 day prior to surgery or those refusing informed consent were excluded. The exact selection process is shown in Fig. 1.

In all study patients, we recorded the occurrence of superficial surgical site infections, organ space abscesses, anastomotic leakage, and their respective therapy in accordance with clinical grading A–C [23] (reoperation (C), radiologic drainage or endoscopic therapy (B), no therapy (A)) as well as postoperative paralytic ileus or infectious diarrhea. The evaluation of the SSI was performed by the treating surgeons.

Follow-up was assured either by patients’ visit in our institution or by a questionnaire that specifically asks for postoperative adverse events in the form of a check list (surgical site infection with specific treatment by their family doctor, surgery-related readmission) to evaluate for 30-day complication rates.

### Operative technique

Our standards in colorectal surgery include a laparoscopic approach using a 4-incision laparoscopic or 6-incision robotic approach. For right hemicolectomy, a mini transverse laparotomy in the right upper quadrant was performed, and for left colonic, sigmoid, or rectal resections, the mini laparotomy in the lower left quadrant of approximately 6 cm is used. For wound edge protection, we use the “Alexis®” Wound Protector system (Applied Medical, Düsseldorf, Germany). For right hemicolectomy, an end-to-end anastomosis with PDS 4-0, extramucosal, continuous suture was performed extracorporeally. The distal resections are performed using the ECHELON FLEX^TM^ laparoscopic stapler and the ILS Circular Stapler (both Ethicon Endosurgery, Johnson & Johnson, Norderstedt, Germany) for a circular stapled anastomosis. In lower rectal resections with total mesorectal excision (TME), we routinely sue diverting ileostomy, but not for partial mesorectal excision (PME). We use easy-flow drains in the vicinity of the anastomosis as indicator drains, which remain in place until the seventh day after surgery. Laparoscopic access sites are closed using intracutaneous sutures with Monocryl (Johnson & Johnson, Norderstedt, Germany) or median laparotomies with staples. We do not place subcutaneous suction drains during wound closure. Peridural catheters or patient-controlled analgesia pumps are used according to patients’ acceptance.

All procedures were performed by the senior author in association with another surgeon from the department. In 2017, we introduced the DaVinci robotic surgery platform to our department. Thus, an increasing number of rectal resections was performed robotically. No other differences in surgical technique or perioperative management occurred between both study periods except for the bowel preparation regime.

### Surgical site infections

A superficial surgical site infection (SSSI) is considered if an operative wound shows signs of local inflammation (purulent drainage, swelling, erythema, local pain), a microorganism can be isolated from the wound fluid, or the wound is opened by the treating physician according to the American Center for Disease Control and Prevention (CDC). We assessed wounds daily during rounds to decide if the wound needs any further treatment. If so, we have a team of wound care specialists who are responsible for the treatment and if needed out-patient follow-up. Wounds are documented in a standard wound protocol that records the size of wounds and wound conditions, e.g., granulation and secretion. If the wound shows a small SSSI and no relevant amount of purulent secretion occurs, no wound swap is taken as it does not influence local therapy. If a local phlegmon is present and the wound discharges purulent fluids, wound swaps are taken for bacterial culture analysis in order to be able to administer targeted antibiotic therapy, but we did not perform routine swaps for study purposes.

Elevated inflammatory parameters, abdominal pain, or other signs of systemic inflammation raise the suspicion of an intraabdominal complication such as intraabdominal abscess or anastomotic leakage (AL). Patients routinely receive CT scans for further evaluation. If there is an intraabdominal abscess, depending on its size and approachability for radiology-guided drain placement, patients receive the appropriate treatment. Only postoperative intraabdominal abscesses that needed further treatment were recorded in the respective complication category. Usually, fluid from abscesses with drain placement is sent for bacterial culture. Patients with rectal resections receive a routine rectoscopy in our department after 7 days to check the anastomosis. As we routinely place a diverting loop ileostomy, endoscopic sponge treatment is initiated, if an AL grade B occurs.

### External validation of study results

After completion of the study analysis, we compared our study results to the external quality control by the German National Reference Center for the Surveillance of Nosocomial Infections (www.nrz-hygiene.de). Here, specific operations, i.e., colorectal resections, are selected according to the OPS code (emergency and elective surgeries). Our own data is collected and validated by our Institute of Hygiene, Hospital Epidemiology, and Environmental Medicine of the Leipzig University Hospital within a follow-up period of 30 days. The data of all treated patients was evaluated independently of their study inclusion. In parallel, the general treatment standard in colorectal surgery was amended according to the study protocol. Thus, the quality control data show the change in nosocomial surgical site infections before and after the start of our study as real-world data. The wound infection rate (WIR) is defined as the number of wound infections in the indicator procedure divided by the count of all indicator procedures multiplied by 100.

### Power calculation

We estimated a case load of approximately 120 patients eligible for study inclusion per year. From the abovementioned literature [1, 24, 25], an odds ratio of 2 to 3 could be expected for the reduction of infectious complications. Subsequently, the number of corresponding pairs would be between 72 and 110 to reach a power of 0.8 with the *a*-error set at 0.05. The power calculation was performed using the open-source software G*power (Düsseldorf, Germany) with assistance from the local Institute for Medical Computer Science, Statistics, and Epidemiology (IMISE).

### Data collection and analysis

Data were collected from the electronic patient charts in the same way for the retrospective and prospective cohort using MS Excel 2016 (Microsoft Corp., Munich; Germany) and analyzed using SPSS 24 (IBM, Ehningen, Germany). For comparison of continuous variables, we used the paired *t*-test, and for discrete variables the McNemar test. For the calculation of the odds ratio (OR) and its respective 95% confidence levels (95% CI), we used the conditional logistic regression. The significance level was set to be *p* = 0.05.

## Results

Between 1 January 2019 and 31 January 2020, a total of 120 patients were found eligible for inclusion. Of those, 19 had to be excluded for various reasons (i.e., no OA received, refusal of informed consent, inability to consent, postponement of surgery). Thus, 101 patients (71 males, 30 females) were included in the study and completed the study protocol. After the completion of the trial period, we performed the matching process in accordance with the before-mentioned parameters. After the primary analysis of the main outcome parameter, i.e., reduction in SSI, we concluded further inclusion of patients as power levels were reached. A total of 92 of those 101 patients were matchable, excluding 5 male and 4 female patients because of the set matching variables’ limits (Fig. 1). The patient characteristics of our intervention group (IG) and the matched control group (CG) are listed in Table 1. The matching process resulted in equally distributed comorbidities.

**Table 1:** Patient characteristics. Values given as mean (± standard deviation) or absolute number (frequency). *BMI* body mass index in kg/m^2^, *CAD* coronary artery disease, *CHF* congestive heart failure, *ASA* American Society of Anesthesiologists performance score

Patient characteristics	Intervention group	Control group	*p*-value
Age	63.17 (±12.8)	62.68 (±12.1)	0.748
Sex (male/female)	66/26	66/26	
BMI	26.04 (±4.9)	26.1 (±4.2)	0.913
Comorbidities			
Hypertension	56 (60.9)	58 (63)	0.864
Peripheral artery disease	3 (3.3)	3 (3.3)	1
CAD/CHF	19 (20.7)	19 (20.7)	1
Diabetes	18 (19.7)	16 (17.4)	0.839
Liver cirrhosis	3 (3.3)	2 (2.2)	1
Malignant disease	70 (76.1)	63 (68.5)	0.118
Chemotherapy	30 (32.6)	26 (28.3)	0.503
Immunosuppression	7 (7.6)	4 (4.3)	0.508
Chronic inflammatory disease	6 (6.5)	5 (5.4)	1
Renal insufficiency	39 (42.2)	45 (48.9)	0.392
Colonization with multidrug-resistant bacteria	7 (7.6)	13 (14,1)	0.263
Preoperative albumin in mg/dl	43.15 (±4.42)	42.59 (±4.67)	0.378
ASA score			
I	1 (1.1)	7 (7.6)	0.046*
II	65 (70.7)	53 (57.6)	
III	26 (28.3)	31 (33.7)	

Most common indications for surgery were colon and rectal cancer as well as sigmoid diverticulitis. This is reflected in the resections performed, with rectal resection (35.1%), sigmoid resection/left colectomy (35.1%), and right colectomy (25%) being the most common interventions (Table 2).

**Table 2:** Indications for surgery and the respective procedures. Values are given as mean (± standard deviation) or absolute number (frequency). *CDC* US Center for Disease Control and Prevention

	Intervention group	Control group	*p*-value
Indications			
Colon cancer	16 (17.4)	19 (20.7)	
Sigmoid cancer	9 (9.8)	8 (8.7)	
Rectal cancer	29 (31.5)	27 (29.3)	
Sigmoid diverticulitis	23 (25)	25 (29.3)	
Polyps of colon	6 (6.5)	4 (4.3)	
Inflammatory bowel disease	2 (2.2)	3 (3.3)	
Peritoneal pseudomyxoma	2 (2.2)	2 (2.2)	
Radiogenic rectal stenosis	1 (1.1)	0	
Sigmoid vesical fistula	2 (2.2)	0	
Anal carcinoma	1 (1.1)	2 (2.2)	
Others	2 (2.2)	2 (2.2)	
Resections			
Right colectomy	23 (25%)	23 (25%)	
Left colectomy	2 (2.2)	2 (2.2)	
Left colectomy/sigmoid	30 (32.6)	30 (32.6)	
Rectal resection	25 (27.5)	25 (27.5)	
Proctocolectomy	2 (2.2)	2 (2.2)	
Hartmann’s reversal	2 (2.2)	2 (2.2)	
Extralevatoric rectal exstirpation	5 (5.4)	5 (5.4)	
Proctocolectomy with pouch	1 (1.1)	1 (1.1)	
Rectal resection with colostomy	2 (2.2)	2 (2.2)	
Surgical approach			
Laparoscopic	79 (85.9)	79 (85.9)	
Open	13 (14.1)	13 (14.1)	
Duration of surgery	243.95 (±106.4)	247.21 (±122.8)	0.739
Wound contamination class (CDC)			
Clean-contaminated (II)	84 (91.3)	79 (85.9)	0.246
Contaminated (III)	8 (8.3)	13 (14.1)	

In the IG, the overall rate of total infections (15.2% vs 30.4%, *p* = 0.013) was significantly reduced with an odds ratio (OR) of 0.333 (95% CI 0.142–0.784; power 0.86) (Table 3). The incidence of superficial SSI (8.7 vs 19.6%, *p* = 0.041) was significantly lower in the IG with an OR of 0.333 (95% CI 0.121–0.917, power 0.74). The CG had predominantly superficial SSI involving the abdominal laparotomy wound (*p* = 0.049). Nonetheless, wound measures did not differ between both groups. With regard to the surgical approach, preoperative bowel decontamination showed a significant decrease in SSI in open surgery (*p* = 0.018), but a less remarkable effect in laparoscopic surgery (*p* = 0.263).

**Table 3:** Postoperative general and infectious complications. *SSI* surgical site infection, *CDAD C. difficile*–associated diarrhea, *AKIN* acute kidney injury

	Intervention group	Control group	*p*-value	Odds ratio	95% CI
Infectious complications					
Total infections	14 (15.2)	28 (30.4)	0.013*	0.333	0.142–0.784
Superficial SSI	8 (8.7)	18 (19.6)	0.041*	0.333	0.121–0.917
Abdominal	5 (5.4)	14 (15.2)	0.049*		
Perineal	1 (1.1)	2 (2.2)			
Anal suture	2 (2.2)	0			
Wounds according to surgical approach					
Laparoscopic	5 (6.3)	9 (11.4)	0.263		
Open	3 (23)	9 (70)	0.018*		
Anastomotic leakage[23]	5 (6.1)	9 (11)	0.549	0.571	0.167–1.952
Grade A	1 (1.1)	1 (1.1)	0.287		
Grade B	3 (3.3)	3 (3.3)			
Grade C	1 (1.1)	5 (5.5)			
Intraabdominal abscess	5 (5.4)	7 (7.6)	0.754	0.714	0.235–2.169
General complications					
Paralysis	10 (10.9)	16 (17.4)	0.26	0.538	0.215–1.350
Diarrhea	5 (5.4)	5 (5.4)	1		
CDAD	1 (1.1)	2 (2.2)	1		
Pneumonia	3 (3.3)	2 (2.2)	1		
Bleeding	4 (4.3)	1 (1.1)	0.375		
Delirium	0 (0)	0 (0)	1		
Cardioembolic	3 (3.3)	0	0.25		
AKIN	12 (13)	15 (16.3)	0.648		
Death	2 (2.2)	1 (1.1)	1		
Readmission due to SSI	3 (3.3)	5 (5.4)	0.688	0.500	0.092–2.730
Postoperative length of stay	12.24 (±10.3)	15.25 (±12.51)	0.057		

Significant (p<0.05)p-values are marked with *

Intraabdominal abscesses (OR 0.714, 95% CI 0.235–2.169) or anastomotic leakage (OR 0.571; 95% CI 0.167–1.952) showed a trend towards a decreased frequency in the IG, but did not reach a significant level. The treatment of anastomotic leakage differed between both groups as only one patient in the IG but five patients in the CG had grade C AL (*p* = 0.287).

Patients with oral bowel preparation with MBP+OA showed a decrease in postoperative paralytic ileus requiring prokinetic medication or nasogastric tube placement, but which also did not reach significant levels (10.9% vs 17.4%; *p* = 0.26; OR 0.538, 95% CI 0.215–1.350).

All other postoperative complications were similar in both groups except for cardioembolic events of which 3 occurred in the IG.

The treatment with preoperative bowel decontamination indicated a shorter hospital stay (LOS) in the IG of about 3 days (12.24 vs 15.25 days; *p* = 0.057).

There was no difference regarding *Clostridioides difficile*–associated diarrhea (CDAD) between IG and CG. No other adverse events were reported with regard to paromomycin or metronidazole application.

### Bacterial and fungal isolates

In the preoperative screening tests for multidrug-resistant organisms (MDRO), we found seven patients being colonized with MDR *Escherichia coli* in the IG compared to eight patients in the CG. In the CG, we also found methicillin-resistant *Staphylococcus aureus* (MRSA) strains, vancomycin-resistant enterococci (VRE, *Enterococcus faecium*), and a MDR *Enterobacter hormaechei* strain (Table 4). As we only collected wound specimens from one wound for microbiological assessment in the IG, we only detected a *Streptococcus agalactiae* strain. In the CG, a wide variety of mostly gram-negative rods and enterococci were detected, with three of them being colonized with *Candida* species. One versus three patients had MDRO in their wound fluid cultures in the IG and the CG, respectively.

**Table 4:** Multidrug-resistant (MDR) bacteria identified by preoperative screening and results of postoperative microbiological cultures. *MRSA* methicillin-resistant *Staphylococcus aureus*, *VRE* vancomycin-resistant *Enterococcus*

	Intervention group	Control group
Preoperative MDR bacteria		
MDR *Escherichia coli*	7 (100)	8 (75)
MRSA	0	2 (16.7)
*VRE Enterococcus faecium*	0	2 (16.7)
*MDR Enterobacter hormachaei*	0	1 (8.3)
Swaps taken	1 (1.1)	12 (13.4)
Swaps sterile	0	1 (8.3)
MDR in wound	1	3
Bacterial isolates		
*Staph. epidermidis*	0	1 (8.3)
*Escherichia coli*	0	5 (41.7)
*Enterococcus faecium*	1 (100)	1 (8.3)
*Enterococcus faecalis*	0	2 (16.7)
*Pseudomonas aeruginosa*	0	3 (25)
*Morganella morgagni*	0	1 (8.3)
*Candida albicans*	0	2 (16.7)
*Proteus mirabilis*	0	1 (8.3)
*Eenterobacter aerogenes*	0	1 (8.3)
*Candida krusei*	0	1 (8.3)
*Streptococcus agalactiae*	1 (100)	0

**Fig. 2:**
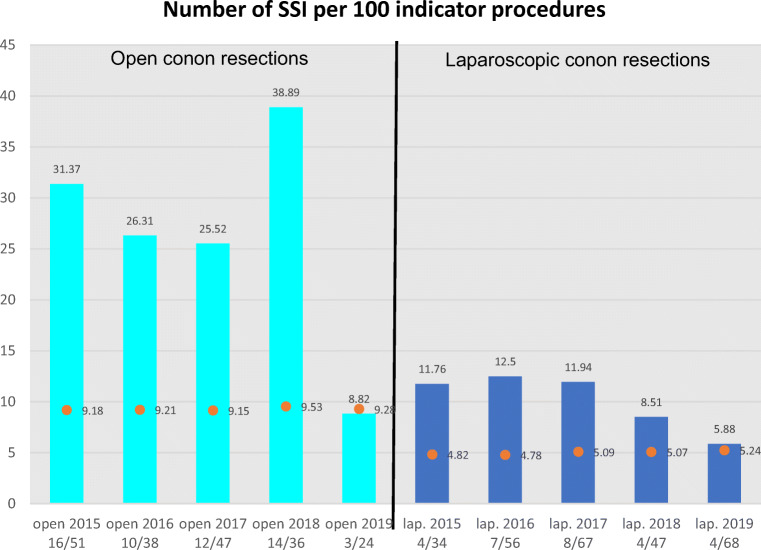
Number of SSI per 100 indicator procedures

The results of our own hospital infection surveillance system, which is monitored by the German National Reference Center, show high nosocomial infection rates prior to the start of our study: 8.51% and 38.89% in the laparoscopic and the open surgery group respectively (see Fig. 2). Since the introduction of our MBP+OA standard, the WIR in open surgery has decreased to 8.2% in open and 5.88% in laparoscopic colon resection.

## Discussion

In this prospective matched case-control study, we sought to evaluate the effects of preoperatively administered oral paromomycin and metronidazole together with mechanical bowel preparation and IV ertapenem to reduce infectious complications in elective colorectal surgery.

Recent literature on preoperative oral antibiotic bowel preparation (OABP) heavily relies on results taken from the ACS-NSQIP registry for different colorectal procedures [5–12]. These studies prefer OABP + MBP as protective regimen with regard to infectious postoperative complications. The recent meta-analysis carried out by Mulder et al. [14] excluded most of the ACS-NSQIP studies to prevent bias by multiple inclusions of the same patients. Nonetheless, they found a benefit of preoperative OABP. Those registry data only take into account if patients received any kind of antibiotic bowel preparation without paying giving any further detail in the specific regimen given. Unfortunately, those data only promote OABP but do not show any superior regimen so that colorectal surgeons cannot use those data as a proper guideline. Furthermore, OABP without MBP has to be taken into consideration, as recent evidence by the ORALEV trial exists, that the mechanical preparation maybe does not exert any additional benefits in reducing the postoperative infectious complications [14, 15].

Internationally, a wide variety of antibiotic regimen can be found. The common combination of an orally available aminoglycoside along with metronidazole, beginning 1 or 2 days preoperatively, is influenced by local drug legislation. For example, several Asian studies report the use of kanamycine [19, 26], whereas the Angloamerican studies mostly have used neomycine [27, 28]. In Germany, most of the studied aminoglycosides do not exist as orally available drug formulations. We therefore chose paromomycin as an orally available aminoglycoside, which has been proven to reduce infectious complications in colorectal surgery used in bowel irrigation preoperatively [22]. Nonetheless, data is scarce on its potential in combination with metronidazole, which is a common adjunct antibiotic in bowel preparation. Metronidazole is an antibiotic drug that especially is effective against anaerobic bacteria such as *Bacteroides* spp. and *Clostridoides* spp., common intestinal bacteria that are isolated from SSI [29]. In our intervention group (IG), we found an OR of 0.333 for total and superficial surgical site infections when taking MBP + OA, which is a similar effect compared to the study by Abis et al. [30], but superior to the results demonstrated by Mulder et al. [25, 31]. Abis et al. performed a proper selective intestinal decontamination by prolonged perioperative administration of the drugs for at least 6 days. Both groups used tobramycin and colistin for 1 and 3 days respectively, but Abis et al. also added amphotericin B as an anti-fungal agent. This raises the question of the need for additional anti-fungal bowel preparation. We found some yeast species in the swaps of the CG but none in the IG. This is most likely due to the small number of swaps taken in the IG. At least all fungi (*Candida albicans* and *Candida krusei*) would have been sensitive to amphotericin B. Unfortunately, the studies using amphotericin B as an oral decontamination agent do not describe wound cultures in order to justify the necessity of addition of anti-fungal agents.

We used ertapenem as a single-shot perioperative IV antibiotic. Other studies routinely used cephalosporines combined with metronidazole. We purposefully chose this first-generation carbapenem to reach a better coverage of the intestinal microbiota including extended-spectrum beta-lactamases (ESBL)–producing enterobacteria combined with the longer plasma half-life of ertapenem of about 4 h [32]. Additionally, ertapenem is effective against most of the gram-negative bacteria that often cause SSI in colorectal surgery (i.e., *Escherichia coli*, *Klebsiella pneumonia*), gram-positive anaerobes, and gram-positive cocci such as *Streptococcus pneumoniae* [29]. The regime of cefuroxime and metronidazole is less effective against those gram-positive and gram-negative anaerobes as well as enterobacterales according to the most recent EUCAST report [33]. Even some *Enterococcus faecalis* strains are susceptible to ertapenem. The only available study comparing different single-shot antibiotic regimen in colorectal surgery found a slightly higher SSI rate using ertapenem compared to cefazoline plus metronidazole (OR 1.48, not significant) [34]. Overall, they attributed the greatest effect in SSI prevention to the oral decontamination and not to IV single-shots. By using ertapenem instead of cefotaxime preoperatively, we could cover all resistant gram-negative bacteria, which we had identified preoperatively by routine screenings. Unfortunately, ertapenem does not cover *Pseudomonas aeruginosa* which may be responsible for AL development under certain circumstances [35]. Still, it seems impossible to cover any possible bacteria with a single-shot antibiotic with the intention to prevent SSI, especially since some of the data on *Pseudomonas aeruginosa* and *Enterococcus faecalis* and their role on anastomotic leakage are in vitro experiments [35, 36]. After all, you have to keep in mind that a responsible administration of reserve antibiotics to prevent bacterial resistance generation is mandatory, and therefore, clinical evaluation of bacterial prevalences in SSI is necessary.

The most pronounced effect of preoperative bowel preparation could be seen in the open surgical group, which was by far the minority of surgical procedures (14%). The respective infection rates were 70% compared to 23% in the laparoscopic surgery group. This is in line with common knowledge on the advantages of laparoscopic surgery. Nevertheless, our results reflect those of Özdemir et al. [21] who evaluated OABP (gentamycine and metronidazole) in open colorectal surgery. They also demonstrated a decrease in SSI from 71 to 36% by using OABP and calculated subsequent savings of about roughly 1700 USD per patient per hospital stay. Similarly, Suzuki et al. [19] found the majority of infections in the open surgical subgroup of their cohort of both open and laparoscopic colorectal cancer surgeries, but they attributed the reduction in infection rates to the addition of mechanical bowel preparation. The SSI rates we found in the CG are relatively high but still in the range of the aforementioned studies. But this elevated rate of SSI stresses the need for measures to improve postoperative patient outcome with regard to infectious complications.

Reduction of anastomotic leakage (AL) has been considered a primary end point in several studies that evaluated preoperative bowel decontamination. Schardey et al. [37] terminated their study after an interim analysis because of a significant reduction in AL in rectal resections. They used a very broad combination of topical antibiotic agents comprising tobramycin, vancomycin, polymyxin B, and amphotericin B versus placebo plus amphotericin B. Their intention was to double cover gram-positive microorganisms after elimination of gram-negative flora. Another randomized study to assess anastomotic leakage in colorectal surgery was recently carried out in the Netherlands [31]. The study was terminated prematurely, as an OR of 0.8 for deep SSI and 0.5 for anastomotic leakage was found. At the point of study cessation, they had included 80 of 966 estimated patients and found 1 versus 2 anastomotic leakages. Hence, it seems doubtful if the authors’ calculated ORs really represent a statistical effect that justifies the termination of the study. Other data hints at reduced AL rates after MBP + OA. Even less aggressive bowel preparation regime (neomycine and metronidazole) could show a reduction in AL in a very recent randomized trial [38]. But almost simultaneously, a different study group published contradictory data with no reduction in AL but slightly lower rates of SSI [39]. We could show a reduced AL rate with less severe AL grades in the IG which did not reach significance levels. The necessary treatment differed remarkably with 5 relaparotomies in the CG versus 1 in the IG, but AL treatment overall did not reach significant levels (*p* = 0.287). This difference is due to the different localization of anastomosis, i.e., rectal versus colonic anastomosis. The detected trend towards a shorter LOS in the IG might also be attributed to the higher rate of reoperation. Although, conservative treatment of an AL (grades A and B) with endoscopic sponge therapy might be even more time-consuming until a sufficient granulation of anastomosis is assured. In total, 10 procedures per group were performed without an anastomosis reducing the subgroup for the analysis of AL. Therefore, a greater cohort is needed to demonstrate a statistically significant effect of our bowel preparation regime with regard to AL. Still, our data taken together with existing data provides diverse recommendations with regard to AL reduction which further stresses the necessity of studies on the best antibiotic regime for perioperative bowel preparation.

Regardless of the statistically significant changes due to the introduction of OA with MBP + OA discovered in our study, the external validation using the national surveillance data of our institution confirms our observations and stresses the importance of MBP + OA.

Our study has several limitations. First and foremost, we conducted a prospective study with matching of a historical control group. The matching process was performed by the authors manually in a blinded fashion from a patient list maintaining the matching variables only. Due to the manual selection pattern, a possible matching bias cannot be excluded. The inclusion of several rare procedures such as proctocolectomy or Hartmann’s reversal might influence the results as well. However, since those patients were properly matched, we do not assume great effects, especially since those procedures consist of the main surgical steps as the more common procedures (i.e., surgical approach, extracorporeal bowel resection, transanal anastomosis, placement of ostomy).

As several studies hint at the benefits of OABP in the prevention of infectious complications in colorectal surgery, future studies should focus on the best antibiotic regimen for preoperative bowel decontamination depending on the microbiological background epidemiology. To date, the inhomogeneity of the regimens used with respect to antimicrobials and drug dosages is still a major problem in the field of OABP.

## Conclusion

Based on the results of our matched-pair analysis, we propose oral paromomycin and metronidazole in single doses for oral antibiotic bowel preparation, paired with a single shot of IV ertapenem preoperatively in colorectal surgery as a promising option to effectively reduce postoperative infectious complications.
